# Management of Non-Culprit Lesions in STEMI Patients with Multivessel Disease

**DOI:** 10.3390/jcm12072572

**Published:** 2023-03-29

**Authors:** Raffaele Piccolo, Lina Manzi, Fiorenzo Simonetti, Attilio Leone, Domenico Angellotti, Maddalena Immobile Molaro, Nicola Verde, Plinio Cirillo, Luigi Di Serafino, Anna Franzone, Carmen Anna Maria Spaccarotella, Giovanni Esposito

**Affiliations:** Department of Advanced Biomedical Sciences, University of Naples Federico II, 80131 Naples, Italy

**Keywords:** non-culprit coronary lesion, STEMI, complete revascularization, multivessel disease, culprit lesion, myocardial infarction

## Abstract

Multivessel disease is observed in approximately 50% of patients with ST-segment elevation myocardial infarction (STEMI) undergoing primary percutaneous coronary intervention (PCI). Data from randomized clinical trials has shown that complete revascularization in the STEMI setting improves clinical outcomes by reducing the risk of reinfarction and urgent revascularization. However, the timing and modality of revascularization of non-culprit lesions are still debated. PCI of non-culprit lesions can be performed during the index primary PCI or as a staged procedure and can be guided by angiography, functional assessment, or intracoronary imaging. In this review, we summarize the available evidence about the management of non-culprit lesions in STEMI patients with or without cardiogenic shock.

## 1. Introduction

The preferred strategy for reperfusion in patients with ST-segment elevation myocardial infarction (STEMI) is primary percutaneous coronary intervention (PCI) [1,2,3,4]. However, approximately 50% of patients presenting with STEMI have multivessel disease, defined as two or more major coronary arteries presenting with obstructive luminal narrowing. Multivessel disease in the STEMI setting is associated with worse outcomes, including a higher risk of mortality, reinfarction, and repeat revascularization [5,6,7]. Factors that may contribute to these poor outcomes include advanced age, multiple cardiovascular risk factors, greater ischemic burden, and left ventricular dysfunction [8]. Additionally, non-culprit lesions (any lesion in the coronary tree not responsible for the acute coronary syndrome) may also be unstable, with similar morphologic characteristics as the culprit lesion [9,10]. With the advancement of coronary devices, the use of new-generation drug-eluting stents [11,12,13], and the systematic adoption of radial access [14], the focus of treatment in STEMI patients has shifted from culprit to non-culprit lesions. Guideline-based recommendations for the management of STEMI patients vary regarding the treatment of non-culprit lesions. The 2017 European Society of Cardiology (ESC) guidelines recommended considering routine revascularization of non-culprit lesions in STEMI patients with multivessel disease before hospital discharge (Class II, level of evidence A) [3], while the 2021 American College of Cardiology/American Heart Association/Society for Cardiovascular Angiography & Interventions (ACC/AHA/SCAI) Guidelines for Coronary Artery Revascularization recommended considering PCI of non-culprit lesions as a planned staged procedure after successful primary PCI in selected patients with STEMI and multivessel disease who are hemodynamically stable (Class I, level of evidence A) [15].

There are many lingering questions regarding the optimal approach to treating non-culprit lesions in STEMI patients. These include when it is best to treat non-culprit lesions (immediately during the index procedure or as a staged procedure) and whether revascularization should be guided by angiographic, functional, or imaging lesion assessment. Each strategy presents pros and cons because angiographic assessment may be overestimated by approximately 10% during the acute STEMI setting [16], whereas changes in the coronary tone and physiology during STEMI may also impact the assessment of non-culprit lesion severity [17,18].

The goal of this review is to summarize and discuss the current literature examining multivessel disease in STEMI patients in order to fully evaluate optimal reperfusion strategies for these patients.

## 2. Angiography-Guided Complete Revascularization

After a few initial trials exploring the feasibility of routine revascularization based on angiography [19,20], the landmark trials contributing to the evidence of an angiography-based approach to complete revascularization (CR) have been the PRAMI [21] (Randomized Trial of Preventive Angioplasty in Myocardial Infarction), CVLPRIT [22] (Complete Versus Lesion-Only Primary PCI Trial), and COMPLETE [23] (Complete versus Culprit-Only Revascularization Strategies to Treat Multivessel Disease after Early PCI for STEMI) trials. The PRAMI trial enrolled 465 patients with STEMI to randomly receive treatment of the culprit lesion (*n* = 231) or CR during the index primary PCI, including the culprit-lesion and all non-culprit lesions with >50% of stenosis evaluated at visual estimation (*n* = 234). In patients randomized to the control group, PCI for angina was recommended only in case of refractory angina with objective evidence of ischemia. The trial was prematurely terminated in view of a significant benefit in the CR group. At a mean follow-up of 23 months, the primary endpoint, a composite of cardiac death, non-fatal myocardial infarction (MI), or refractory angina, occurred in 9% and 23% of patients randomized to complete vs. culprit-only revascularization (hazard ratio (HR) 0.35; 95% confidence intervals (CI): 0.21 to 0.58; *p* < 0.001). Of interest, cardiac mortality (HR 0.34, 95% CI: 0.11–1.08), the risk of reinfarction (HR 0.32, 95% CI: 0.13–0.75), and refractory angina (HR 0.35, 95% CI: 0.18–0.69) were decreased by CR [21].

In the CvLPRIT trial, CR was based on the angiographic finding of stenosis in the non-culprit lesion > 70% (or ≥50% stenosis in two different angiographic views). At variance with the PRAMI, CR in the CvLPRIT trial was allowed during the index PCI or hospitalization. Overall, a total of 296 patients with STEMI were enrolled. At the 1-year follow-up, the risk of the primary endpoint, a composite of all-cause death, recurrent MI, heart failure, or ischemia-driven revascularization (IDR), was significantly reduced in patients randomized to complete vs. culprit-only revascularization (10% vs. 21.2%; HR 0.45, CI: 0.24–0.84; *p* = 0.009) [22]. Although the trial was not powered for individual endpoints, each component of the primary endpoint was numerically reduced in patients undergoing CR. 

The largest trial comparing CR vs. culprit-only revascularization in STEMI patients was the COMPLETE trial [23] that enrolled a total of 4041 patients. Non-culprit lesions were considered significant when at >70% with visual angiographical estimation or between 50% and 69% with an FFR (fractional flow reserve) < 0.80. Because FFR was performed only in 0.8% of patients randomized to the experimental arm, the trial could be classified as an angiography-guided strategy. Non-culprit lesion PCI was performed during the index hospitalization (but not during the index procedure) or in a staged PCI within 45 days from the randomization, a choice that was made by investigators prior to the randomization. At the 3-year follow-up, the first coprimary endpoint, a composite of CV death or new MI, occurred in 7.8% in the CR group vs. 10.5% in the culprit-only group (HR 0.74, *p* = 0.004). This result was driven by a lowered risk of reinfarction in the CR group. The second coprimary endpoint, a composite of CV death, new MI, or IDR, occurred in 8.9% of the CR group vs. 16.7% of the culprit-only group (HR 0.51, *p* < 0.001). Among the patients assigned to the CR group, one third had the second procedure after hospital discharge within 45 days from the randomization (*n* = 663, with a median time of 23 days for the second PCI) and the remaining two thirds had the second PCI during the index admission (*n* = 1353, with a median time of 1 day for the second PCI) [23]. A prespecified subgroup analysis about the timing of revascularization and the time course of the benefit of CR was performed [24]. In the group of patients with intended non-culprit revascularization during index hospitalization, the incidence of the first coprimary outcome was 2.7% per year in patients randomized to CR vs. 3.5% per year in those with culprit lesion-only revascularization (HR: 0.77; 95% CI: 0.59 to 1.00). In the group of patients with intended CR after discharge, the rate of first coprimary endpoint in the CR group versus the culprit lesion-only revascularization group was 2.7% per year vs. 3.9% per year, respectively (HR: 0.69; 95% CI:0.49 to 0.97). Similar results were observed for the second coprimary endpoint in the two temporal strata of both revascularization groups: in the stratum of patients with intended non-culprit revascularization during index hospitalization, the incidence was 3.0% per year vs. 6.6% per year (HR: 0.47; 95% CI: 0.38 to 0.59); in the group of patients with intended non-culprit revascularization after discharge, the incidence was 3.1% per year vs. 5.4% per year, respectively (HR: 0.59; 95% CI: 0.43 to 0.79). In a landmark analysis at 45 days, the benefit of CR remained preserved if PCI of non-culprit lesions was performed within 45 days after the index PCI [24]. 

In a substudy of the COMPLETE trial evaluating the severity of the non-culprit lesion using quantitative coronary angiography [25], the rate of the first coprimary endpoint (the composite of CV death or new MI) was reduced in patients with stenosis equal or greater than 60% at quantitative coronary angiography (2.5% per year in patients randomized to CR vs. 4.2% per year in patients randomized to culprit lesion-only revascularization (HR: 0.61; 95% CI: 0.47 to 0.79), whereas there were no differences in patients with a stenosis less than 60% (3.0% per year in patients randomized to CR vs. 2.9% per year in patients randomized to culprit lesion-only revascularization (HR: 1.04; 95% CI: 0.72 to 1.50). Although for the second coprimary endpoint a significant benefit was found in both subgroups, the magnitude of HR reduction was greater in patients with more severe stenoses [25].

## 3. Function-Guided Complete Revascularization

Microvascular dysfunction and vasospasm during STEMI may alter physiological assessment of non-culprit lesions. The accuracy of FFR to evaluate non-culprit lesions during primary PCI was first assessed in a prospective study including 101 patients [26]. FFR measurements in 112 non-culprit lesions were obtained immediately after PCI of the culprit lesion and were remeasured within three months. FFR values in non-culprit lesions did not change significantly between the acute and late phases after MI. In a subsequent study including 73 patients with STEMI, FFR values during the index procedure were significantly higher than those observed at follow-up, especially in patients with larger infarcts [17]. These findings raised concerns about the reliability of FFR to assess the severity of non-culprit lesions during the same hospitalization as the primary PCI.

### 3.1. FFR-Guided Complete Revascularization

The DANAMI-3-PRIMULTI trial [27] (complete revascularization versus treatment of the culprit lesion only in patients with ST-segment elevation myocardial infarction and multivessel disease) was the first randomized trial employing a staged FFR-based PCI of non-culprit lesions after successful PCI of the culprit lesion. A total of 627 patients was randomly allocated to either no further invasive treatment or complete FFR-guided revascularization within two days after the primary PCI in case of stenosis > 90% or >50% and FFR < 0.80. CR guided by FFR significantly reduced the risk of the primary endpoint, a composite of all-cause mortality, non-fatal reinfarction, or IDR of non-culprit lesions at 27 months, compared with no further invasive intervention after the primary PCI (13% vs. 22%, HR 0.56, 95% CI: 0.38–0.83; *p* = 0.004) [27]. This result was mainly driven by fewer repeat revascularizations, whereas the risk of all-cause mortality and non-fatal reinfarction did not differ between the two groups. However, in view of the modest sample size, the trial was not powered to detect significant differences in clinically relevant outcomes. Moreover, as the trial proved only a reduction in the risk of subsequent revascularization not accompanied by a lowered risk of death or reinfarction, it was uncertain how these results would impact clinical practice. Of note, in a substudy of the same trial, the benefit from function-guided CR was greater among patients with multivessel disease [28]. However, even in this high-risk subgroup, there was no significant reduction in the prognostically relevant outcomes of all-cause mortality or non-fatal reinfarction. A subsequent larger trial, the COMPARE-ACUTE trial (Fractional Flow Reserve–Guided Multivessel Angioplasty in Myocardial Infarction), randomized 885 multivessel patients to FFR-guided PCI of non-culprit lesions during the index procedure versus culprit lesion-only PCI [29]. At 1 year, the composite endpoint of all-cause mortality, non-fatal MI, any revascularization, or cerebrovascular event was significantly reduced in patients randomized to FFR-guided CR (8% vs. 21% HR: 0.35; 95% CI: 0.22–0.55; *p* < 0.001). Notably, there was no significant difference between the two study groups in the risk of death or MI, and the benefit for the primary composite endpoint was principally attributed to a reduction in the risk of any revascularization in the FFR group. Among patients undergoing CR, the physiology assessment required only a few additional minutes to the total procedural time, which may be important to avoid additional catheterization procedures. Of interest, treatment allocation and angiographic findings of the non-culprit lesions were not blinded to the operators, and this may have introduced a bias triggering new unplanned revascularization procedures in the control arm. A substantial difference between the two trials was the timing of CR required in the staged procedure for the DANAMI-3-PRIMULTI trial and during the index procedure for the COMPARE-ACUTE trial [27,29]. A meta-analysis including the DANAMI-3-PRIMULTI and COMPARE-ACUTE trials showed that FFR-guided multivessel PCI may also lead to a reduction of urgent revascularization [30]. In a recent meta-analysis of ten trials, including more than 7000 patients, CR was associated with a reduced risk of CV death compared with culprit lesion-only PCI, with no difference whether it was achieved using FFR assessment or not [31].

### 3.2. Angiography-Guided vs. FFR-Guided Revascularization 

A total of 1171 patients with STEMI and multivessel disease were randomized in the FLOWER-MI trial (FLOW Evaluation to Guide Revascularization in Multi-vessel ST-elevation Myocardial Infarction) to CR by means of an FFR- or angiography-guided strategy (angiography > 50% in the control group and angiography > 50% and FFR < 0.80 in the intervention group) [32]. An FFR-guided strategy was not associated with a decreased risk of death, MI, or urgent revascularization at 1 year and carried a non-significantly higher risk of MI in the FFR group. However, the findings should be interpreted with caution due to the wide confidence intervals for the hazard ratio for the primary endpoint, which were compatible with either a 22% relative benefit or 123% relative harm associated with the FFR-guided strategy [32]. 

Recently, in the FRAME-AMI trial (FFR Versus Angiography-Guided Strategy for Management of AMI With Multivessel Disease), a total of 562 patients were randomly assigned to either FFR-guided PCI (FFR ≤ 0.80) or angiography-guided PCI (stenosis of >50%) for non-culprit lesions. Among them, 60% underwent CR and 40% were treated by a staged procedure during the same hospitalization. PCI was performed for non-culprit lesions in 64.1% of the FFR-guided PCI group and 97.1% of the angiography-guided PCI group. The risk of the primary endpoint, a composite of time to death, MI, or repeat revascularization, was significantly reduced in the FFR-guided PCI group vs. the angiography-guided PCI group (7.4% vs. 19.7%; HR: 0.43; 95% CI: 0.25–0.75; *p* = 0.003) at the 3.5-year follow-up. The benefit of FFR-guided PCI on the primary endpoint was consistent regardless of STEMI or non-STEMI patients [33].

## 4. Imaging-Guided Complete Revascularization

In the setting of STEMI, plaque instability may not be confined to the culprit lesion only [34]. Therefore, intravascular imaging has a key role in multivessel STEMI patients, given its ability to detect features of vulnerability even in non-obstructive lesions potentially responsible for future acute thrombotic events [35,36,37,38,39]. Different catheter-based imaging techniques include intravascular ultrasound (IVUS), optical coherence tomography (OCT), and near-infrared spectroscopy (NIRS). OCT is the most reliable technique in assessing the thickness of the fibrous cap, plaque components, and eventual macrophage infiltration given its high spatial resolution (10-fold higher than IVUS). In the CLIMA trial (Relationship between coronary plaque morphology of the left anterior descending artery and 12 months clinical outcome) including a total of 1776 lesions, four OCT plaque features were associated with a higher risk of coronary events: the presence of a minimal lumen area (MLA) < 3.5 mm^2^, fibrous cap thickness < 75 mm, lipid arc circumferential extension > 180°, and OCT-defined macrophage infiltration. Of note, the simultaneous presence of these features was observed in 18.9% of patients experiencing the composite primary endpoint of target-segment MI and/or cardiac death [39]. In a substudy of the COMPLETE trial, OCT of at least 2 coronary arteries before non-culprit lesion PCI was performed in 93 patients [40]. Thin cap fibroatheroma (TCFA) was detected in the non-culprit lesions of about half of the patients and were more commonly found in obstructive lesions, defined as >70% visual diameter stenosis, than in non-obstructive lesion. This may explain the net clinical benefit of CR when compared with culprit-only revascularization, corroborating a strategy of routine PCI of non-culprit lesions in this subset of patients [40,41]. These findings were similar to those of Tian et al. who found a two-fold increase of TCFA detection and more features of plaque vulnerability in non-culprit angiographically severe stenosis in 643 plaques from 255 subjects who underwent three-vessel OCT imaging [42].

Different from OCT, IVUS has a higher penetration depth, allowing for a better assessment of the plaque burden. In the PROSPECT trial (a prospective natural-history study of coronary atherosclerosis), a plaque burden ≥70%, the presence of a TCFA, and an MLA ≤ 4 mm^2^ emerged as independent predictors of adverse events in non-culprit lesions among 697 patients with ACS undergoing three-vessel virtual histology (VH)-IVUS. Lesions responsible for adverse events at follow-up were frequently angiographically mild [35]. The detection of lipid-rich plaques may be facilitated using NIRS, as shown in the Lipid-Rich Plaque and ATHEROREMO-NIRS studies [36,37]. An increase in the maximum lipid core burden index was responsible for a higher risk of non-culprit Major Adverse Cardiac Events (MACE), including cardiac death, cardiac arrest, non-fatal MI, ACS, revascularization, and readmission to hospital for angina at the 2-year follow-up [36,37]. Non-culprit lipid-rich plaques were associated with an increased risk of MACE at the 2-year follow-up also in the Massachusetts General Hospital OCT registry [38]. However, it must be recognized that plaque vulnerability features have demonstrated highly negative predictive value but low positive predictive value. Therefore, their clinical significance remains to be fully addressed [35]. The FORZA trial (Fractional Flow Reserve or Optical Coherence Tomography to Guide Management of Angiographically Intermediate Coronary Stenosis) compared OCT guidance and FFR guidance in patients with angiographically intermediate coronary lesions and showed that an OCT-guided approach may reduce the risk of adverse events, at the expense of higher costs [43].

These findings suggest that a morphological approach to PCI in high-risk patients is feasible and can provide more specific treatment compared with a standard approach, which presents the limitation of lack of information about plaque characteristics. 

### Imaging Guidance for Percutaneous Coronary Intervention

The use of intracoronary imaging provides an accurate assessment of lesion length and vessel size. For the stent size, both OCT and IVUS use the external elastic lamina, usually resulting in selection of a larger stent compared to a lumen-based approach [44]. Landing zones within an area of residual plaque burden > 50% and within a lipid pool should be avoided given the association with stent restenosis (SR) and an increased risk of peri-procedural MI [45,46]. When assessing post-stenting results, the recommended target stent expansion is >80%, with a minimal stent area > 5.5 mm^2^ with IVUS or >4.5 mm^2^ with OCT for non-left main lesions; dissections increasing adverse events are usually defined as involving deeper layers (medial or adventitia), having extensive lateral (>60°), and longitudinal extension (>2 mm), and localized at the distal edge [45].

## 5. Complete Revascularization in Cardiogenic Shock

Cardiogenic shock (CS) affects up to 10% of patients with acute myocardial infarction and continues to be associated with a high mortality and morbidity [47]. In patients with acute MI complicated by CS, an immediate revascularization strategy is associated with a mortality reduction [48]. Consistently, in current ESC guidelines focused on myocardial revascularization, when coronary anatomy is favorable, immediate PCI of the culprit lesion is indicated for patients with CS due to acute MI, independent of time delay of symptom onset [49]. 

In the SHOCK trial (The Should We Emergently Revascularize Occluded Coronaries for Cardiogenic Shock), 302 patients developing CS after acute MI were randomized to emergency PCI versus initial medical stabilization. At six months, all-cause mortality was significantly lower in the group assigned to revascularization than in the group assigned to medical therapy [50].

A subsequent analysis of the SHOCK trial showed that there was no difference in 30-day or 1-year survival between patients treated with PCI and those treated with coronary bypass artery grafting (CABG) [51]. Although emergency CABG is not representative of contemporary management of revascularization in patients with CS, the benefits associated with CABG might be related to the protection of the ischemic myocardium with cardioplegia, ventricular unloading during cardiopulmonary bypass, and revascularization of non-infarct zones. CS is a high-acuity, complex, and hemodynamically diverse state of end-organ hypoperfusion that is frequently associated with multivessel disease, which is more frequently followed by a worse outcome and mortality than single-vessel disease. The current American College of Cardiology/American Heart Association (ACC/AHA) and ESC guidelines do not recommend PCI of non-IRA at the time of primary PCI during acute myocardial infarction in patients with CS (class III, level of evidence B) because of the higher risk of death or renal failure [15,16,17,18,19,20,21,22,23,24,25,26,27,28,29,30,31,32,33,34,35,36,37,38,39,40,41,42,43,44,45,46,47,48,49]. Risks associated with CR include volume overload, contrast renal injury, more prolonged procedure times, and ischemic complications in the non-culprit vessel, resulting in further hemodynamic deterioration. PCI of non-culprit lesions may cause coronary flow alterations due to the embolization of plaque that impairs the collateral blood supply to the infarct zone.

The CULPRIT-SHOCK [52] (Culprit Lesion Only PCI Versus Multivessel PCI in Cardiogenic Shock) trial showed superior outcomes for culprit lesion-only versus immediate multivessel revascularization in patients presenting with acute myocardial infarction, multivessel disease, and CS. A meta-analysis by Khan et al., which included 12 observational studies and 2 trials (CULPRIT-SHOCK trial and Intra-Aortic Balloon Pump (IABP) trial), showed that multivessel PCI was not associated with increased mortality when compared with culprit lesion-only PCI, but showed higher rates of acute kidney injury (AKI) [53]. This could potentially be explained by the higher contrast volume used in multivessel PCI. Although the rates of rehospitalization for heart failure and repeat revascularization were higher in the culprit lesion-only revascularization group, mortality did not differ significantly between the two groups at one year. A post-hoc analysis of the CULPRIT-SHOCK trial showed that culprit lesion PCI vs. multivessel PCI resulted in a significantly lower risk of death and kidney replacement therapy at 30 days (45.6% vs. 63.5%; *p* = 0.007) and of mortality at 1 year (50% vs. 69.6%; *p* = 0.003) only in patients with a culprit lesion of the left main or proximal left anterior descending artery [54]. It is important to note the limitations of the trial in which only 28% of patients received a mechanical circulatory support device, including that 12% of the total cohort were supported with the transvalvular axial flow device Impella (Abiomed, Danvers, MA, USA).

## 6. Treatment Algorithm

Approximately 50% of patients undergoing primary PCI for STEMI have multivessel coronary artery disease [55]. The strategy of CR was associated in many studies with a lower risk for MACE, driven mainly by a lower rate of MI and repeat revascularization [21,22,23,27,29].

PCI options for these patients include: (1) primary PCI of the culprit lesion followed by PCI of non-culprit lesions only for documented ischemia on invasive tests or intermediate or high-risk characteristics on pre-discharge non-invasive testing; and (2) primary PCI of culprit arteries followed by routine PCI of non-culprit lesions.

Non-culprit lesions may be stable plaques, for which revascularization could not give any benefit, or unstable plaques, which confer a higher risk of rupture and then of future cardiovascular events, thus routine non-culprit lesion PCI could be beneficial [27]. The decision about which approach to use has been matter of debate and what is the best choice to determine the significance of non-culprit lesions remains controversial. The question of whether the hemodynamic relevance of non-culprit lesions may be determined by invasive physiological assessments or by non-invasive ischemia imaging remains unresolved, but irrespective of the employed method, the general purpose should be to treat only hemodynamically relevant lesions [56,57]. Finally, the optimal timing for staged procedures also remains uncertain. Current STEMI guidelines recommend CR before discharge, but they were written before the COMPLETE trial results [23], in which the reduction in MACE in the CR group occurred irrespective of the time (0–45 days) of non-culprit PCI, emphasizing that CR may also be postponed to elective admission [24]. Only for patients presenting with cardiogenic shock, based on the data from the CULPRIT-Shock trial, current guidelines no longer recommend a routine CR during the index procedure [52].

**Figure 1:**
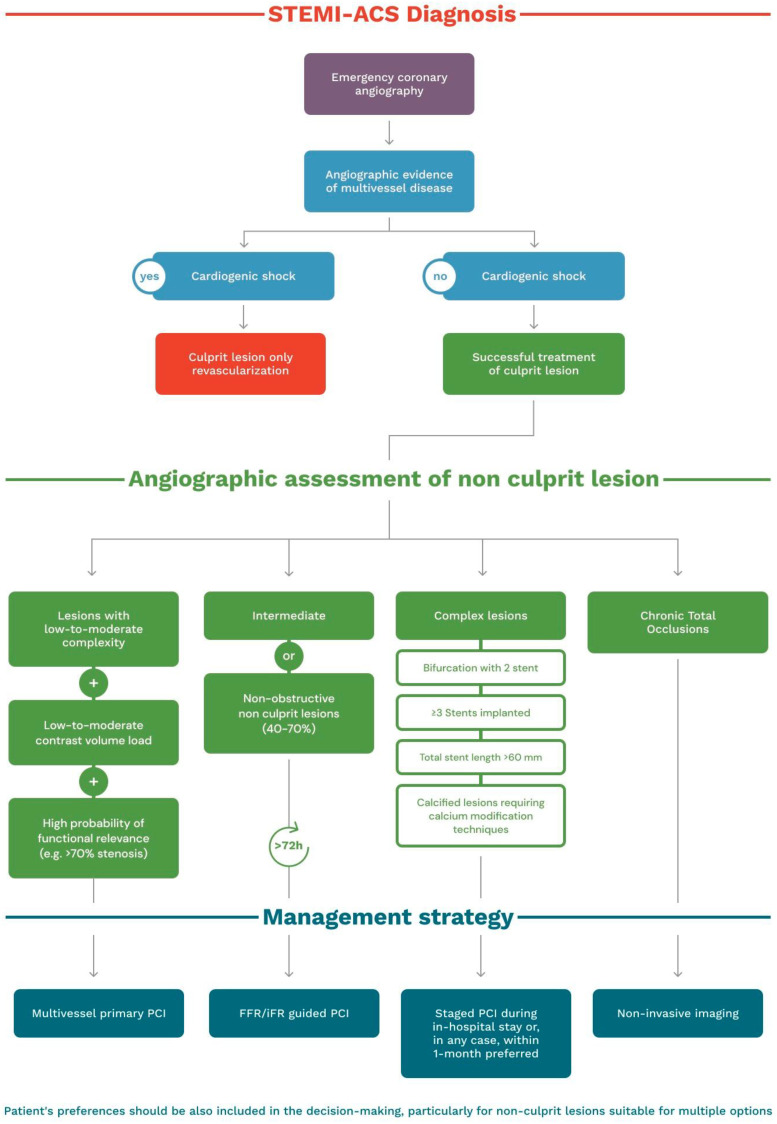
Treatment algorithm.

The results of the ongoing trials iMODERN (ClinicalTrials.gov Identifier: NCT03298659), FULL-REVASC (ClinicalTrials.gov Identifier: NCT02862119), SAFE-STEMI (ClinicalTrials.gov Identifier: NCT02939976), and BIOVASC (ClinicalTrials.gov Identifier: NCT03621501) are awaited to establish the optimal strategy in patients with STEMI and multivessel disease. Meanwhile, we propose a pragmatic algorithm to better manage these patients given the current evidence (Figure 1). Involving patients in the decision-making process for non-culprit lesions in the STEMI setting is key, particularly when immediate and staged treatment are both viable options. By engaging patients in the decision-making process, clinicians can ensure that the treatment plan aligns with the patient’s goals, values, and preferences, possibly leading to better health outcomes and improved patient satisfaction. Shared decision-making can also improve patient engagement and adherence to the chosen treatment plan. Therefore, it is important for clinicians to prioritize patient involvement in the decision-making process when treating non-culprit lesions in the STEMI setting.

In patients without cardiogenic shock, decision-making should be related to patient and anatomic characteristics. The first goal of primary PCI is and should remain the swift restoration of the coronary blood flow in the culprit artery. In patients with successful treatment of the culprit lesion, multivessel primary PCI should be considered for lesions with low-to-moderate complexity, after low-to-moderate contrast volume load, and in the presence of high-degree stenoses with high probability of functional relevance (e.g., >70% stenosis subtended by a relatively large area of myocardium). Patients with intermediate or non-obstructive non-culprit lesions (40–70%) can be managed with an FFR-guided strategy. In the case of complex lesions (e.g., bifurcation with 2 stent, ≥3 stents implanted, total stent length > 60 mm, calcified lesions requiring calcium modification techniques), a staged PCI during in-hospital stay or, in any case, within 1 month should be preferred. As the evidence from randomized trials did not include patients with chronic total occlusions, a diagnostic integration with non-invasive imaging should be considered for staged PCI procedures. Finally, patient preferences should be also included in decision-making, particularly for non-culprit lesions suitable for multiple options (Table 1).

## Figures and Tables

**Table 1:** Randomized controlled trials on culprit lesion-only PCI versus multivessel PCI. ACS, acute coronary syndrome; CR: complete revascularization; FFR: fractional flow reserve; FU: follow-up; IDR: ischemia driven revascularization; MACE: major adverse cardiovascular events; MI: myocardial infarction; NA, not available; NCL: non-culprit lesion; PCI: percutaneous coronary intervention; RR, repeat revascularization.

Study	Population (n), Randomization Ratio	Intervention Group	Control Group	Assessment of NCL	Primary Endpoint	Results
***PRAMI***	*n* = 465, 1:1	234 patients with CR during index procedure	231 patients with culprit-only revascularization	Angiography > 50%	MACE: Cardiovascular death, non-fatal MI, refractory angina at 23 months FU.	9% vs. 23% (*p* < 0.001)
***CvLPRIT***	*n* = 296, 1:1	150 patients with CR during index procedure or index admission	146 patients with culprit-only revascularization	Angiography > 70% (1 view) or >50% (2 views)	MACE: Death, MI, any repeat revascularization, HF at 1-year FU.	10% vs. 21.2% (*p* = 0.009)
***COMPLETE***	*n* = 4041, 1:1	2016 patients with CR during staged procedure in index admission or post-discharge	2025 patients with culprit-only revascularization	Angiography > 70% or angiography between 50%–69% and FFR < 0.80	(1) Composite of cardiovascular death and MI. (2) Composite of cardiovascular death, MI, and ischemia driven revascularization (IDR) at 3-year FU.	(1) 7.8% vs. 10.5% (*p* = 0.004) (2) 8.9% vs. 16.7% (*p* < 0.001)
***DANAMI-3-PRIMULTI***	*n* = 627. 1:1	314 patients with CR during staged procedure in index admission	313 patients with culprit-only revascularization	Angiography > 90% or angiography > 50% and FFR < 0.80	MACE: Death, re-infarction, ischemia driven revascularization at 27-month FU.	13% vs. 22% (*p* = 0.004)
***COMPARE-ACUTE***	*n* = 885, 2:1	295 patients with CR during index procedure or index admission	590 patients with culprit-only revascularization	Angiography > 50% and FFR < 0.80	MACE: Death, non-fatal MI, revascularization, cerebrovascular events at 1-year FU.	8% vs. 21% (*p* < 0.001)
***FLOWER-MI***	*n* = 1163, 1:1	586 patients with CR FFR-guided during index procedure or index admission	577 patients with CR angio-guided during index procedure or index admission	Angiography > 50% in the control group and angiography > 50% and FFR < 0.80 in the intervention group	Composite of death for any cause, non-fatal MI, and unplanned hospitalization leading to urgent revascularization at 1-year FU.	5.5% vs. 4.2% (*p* = 0.31)
***FRAME AMI***	*n* = 562, 1:1	284 patients with CR FFR-guided PCI	278 patients with CR angio-guided PCI	Angiography > 50% in the control group Angiography > 50% and FFR < 0.80 in the intervention group	Composite of time to death, MI or RR at 3.5-year FU.	7.4% vs. 19.7% (*p* = 0.003)
***CULPRIT-SHOCK***	*n* = 706, 1:1	344 patients with CS in culprit lesion-only PCI	342 patients with CS in immediate multivessel PCI	Angiography > 70%	Composite of death or severe renal failure leading to renal replacement therapy at 30-day FU.	45.9% vs. 55.4 (*p* = 0.01)

## Data Availability

Not applicable.

## Abbreviations

ACC	American College of Cardiology
ACS	acute coronary syndrome
AHA	American Heart Association
AKI	acute kidney injury
CABG	coronary bypass artery grafting
CI	confidence interval
CR	complete revascularization
CS	cardiogenic shock
CV	cardiovascular
ESC	European Society of Cardiology
FFR	fractional flow reserve
FU	follow-up
HR	hazard ratio
IDR	ischemia-driven revascularization
IRA	non-infarct related artery
IVUS	intravascular ultrasound
MACE	major adverse cardiac events
MI	myocardial infarction
MLA	minimal lumen Area
NIRS	near-infrared spectroscopy
OCT	optical coherence tomography
PCI	percutaneous coronary intervention
QCA	quantitative coronary angiography
RR	repeat revascularizations
SCAI	Society for Cardiovascular Angiography & Interventions
SR	stent restenosis
STEMI	ST-segment elevation myocardial infarction
TCFA	thin cap fibroatheroma
VH	virtual histology
